# Dual transcriptome analysis reveals insights into the response to *Rice black-streaked dwarf virus* in maize

**DOI:** 10.1093/jxb/erw244

**Published:** 2016-07-18

**Authors:** Yu Zhou, Zhennan Xu, Canxing Duan, Yanping Chen, Qingchang Meng, Jirong Wu, Zhuanfang Hao, Zhenhua Wang, Mingshun Li, Hongjun Yong, Degui Zhang, Shihuang Zhang, Jianfeng Weng, Xinhai Li

**Affiliations:** ^1^Institute of Crop Science, Chinese Academy of Agricultural Sciences, Zhongguancun South Street, Haidian District, Beijing 100081, China; ^2^College of Agronomy, Northeast Agricultural University, Mucai Street, XiangFang District, Harbin, Heilongjiang Province 150030, China; ^3^Jiangsu Academy of Agricultural Sciences, Zhongling Street, Xuanwu District, Nanjing, Jiangsu Province 210014, China

**Keywords:** Degradome, maize, miRNA, *Rice black-streaked dwarf virus*, transcriptome, virus-response.

## Abstract

Maize rough dwarf disease (MRDD) is a viral infection that results in heavy yield losses in maize worldwide, particularly in the summer maize-growing regions of China. MRDD is caused by the *Rice black-streaked dwarf virus* (RBSDV). In the present study, analyses of microRNAs (miRNAs), the degradome, and transcriptome sequences were used to elucidate the RBSDV-responsive pathway(s) in maize. Genomic analysis indicated that the expression of three non-conserved and 28 conserved miRNAs, representing 17 known miRNA families and 14 novel miRNAs, were significantly altered in response to RBSDV when maize was inoculated at the V3 (third leaf) stage. A total of 99 target transcripts from 48 genes of 10 known miRNAs were found to be responsive to RBSDV infection. The annotations of these target genes include a *SQUAMOSA* promoter binding (SPB) protein, a P450 reductase, an oxidoreductase, and a ubiquitin-related gene, among others. Characterization of the entire transcriptome suggested that a total of 28 and 1085 differentially expressed genes (DEGs) were detected at 1.5 and 3.0 d, respectively, after artificial inoculation with RBSDV. The expression patterns of cell wall- and chloroplast-related genes, and disease resistance- and stress-related genes changed significantly in response to RBSDV infection. The negatively regulated genes GRMZM2G069316 and GRMZM2G031169, which are the target genes for miR169i-p5 and miR8155, were identified as a nucleolin and a NAD(P)-binding Rossmann-fold superfamily protein in maize, respectively. The gene ontology term GO:0003824, including GRMZM2G031169 and other 51 DEGs, was designated as responsive to RBSDV.

## Highlight

Infection by *Rice black-streaked dwarf virus* (RBSDV) causes heavy yield losses in maize. We conducted analyses of microRNAs, the degradome, and transcriptome sequences in order to gain insights into RBSDV-responsive pathway(s) in maize.

## Introduction

Maize rough dwarf disease (MRDD) is a viral disease of maize that occurs worldwide, but can be particularly severe in the maize-growing regions of China (Meng *et al.*, 2008; Su *et al.*, 2008; Zhang, 2010). Yield losses of 30–100% due to widespread severe infections occurred in the Yellow and Huai River valleys in China between 2008 and 2012 (Meng *et al.*, 2008; Su *et al.*, 2008; Zhang, 2010; Tao *et al.*, 2013*a*; Lu *et al.*, 2014; ). Symptoms of MRDD typically include dwarfed plant height, malformed tassels with no pollen, small ears or failure of heading, and green leaves that appear rough due to waxy enations (Tao *et al.*, 2013*a*,b).

In China, MRDD is thought to be caused by *Rice black-streaked dwarf virus* (RBSDV), which is transmitted in a persistent manner by the small brown planthopper (SBPH, *Laodelphax striatellus*) (Zhang *et al.*, 2001). RBSDV belongs to the genus *Fijivirus* in the family *Reoviridae*, and is composed of 10 genomic double-stranded RNA (dsRNA) segments (S1–S10), which encode 13 proteins (Milne and Lovisolo, 1977; Wang *et al.*, 2003). The pathogenic pathway involving the functions of the virus proteins and interaction proteins has been partially determined in rice and maize. For example, P5-2 (encoded by the second open reading frame, ORF, of S5) is a non-structural protein that can be specifically targeted to chloroplasts (Liu *et al.*, 2015). P7-2 (encoded by the second ORF of S7) might be a viral F-box protein that is referred to the ubiquitination pathway during the interaction with plant SKP1 (a core subunit of SCF ubiquitin ligase) (Wang *et al.*, 2013).

Several microRNAs (miRNAs) that are related to disease symptoms are known to be affected by viral infection in plants. In previous studies, the expression of miRNAs 156, 160, 164, 166, 169, and 171 were found to be altered in a manner correlated with the symptoms of *Tobacco mosaic virus* (TMV) disease in *Nicotiana tabacum* (*N.t.*) (Bazzini *et al.*, 2007). In addition, P1/HC-Pro, which is the *Turnip mosaic virus* (TuMV)-encoded RNA-silencing suppressor, interfered with the activity of miR171 (also known as miRNA39) by suppressing RNA silencing during the infection of Arabidopsis with TuMV (Kasschau *et al.*, 2003). *ARGONAUTE1* (*AGO1*) mRNA levels are reduced when endogenous miR168 expression is induced by viruses infecting plants (Varallyay *et al.*, 2010). In subsequent work, when the RBSDV-responsive miRNAs and the transcriptome were characterized in rice leaves and roots from healthy rice plants or those infected with RBSDV, the expression of 14 miRNAs in leaves and 16 miRNAs in roots changed significantly. A total of 104 target transcripts were responsive to RBSDV infection 1 month post-infection, when RBSDV-inoculated rice began to show symptoms of rice black-streaked dwarf disease (RBSDD) (Sun *et al.*, 2014).

During the response to *Rice stripe virus* (RSV), the expression of genes for peroxidase biosynthesis, leucine-rich repeat (LRR) receptor-like protein kinase, a pathogenesis-related protein, a glycine-rich cell wall structural protein, xyloglucan hydrolase, and cellulose synthase-related genes were affected in rice (Zheng *et al.*, 2013). The expression profile of plants with MRDD demonstrates that the expression of various disease resistance-related genes, cell wall synthesis genes, and development-related genes are dramatically altered at the stage when white streaks appear on the topmost newly expanded leaf (Jia *et al.*, 2012). Maize can be infected by RBSDV at any growth stage, but infections at the seedling stage can be especially serious. In spite of this, a combined analysis of the miRNA-degradome and the transcriptome has not previously been reported for responses to RBSDV infection during early growth stages of maize.

In order to investigate the miRNA-regulated network response to RBSDV during early stages of infection, deep sequencing of miRNAs, the degradome, and the transcriptome were performed to determine which genes are the targets of specific miRNAs, and to elucidate the molecular pathway of RBSDV-infection in maize plants. Elucidation of the molecular response to RBSDV infection was achieved by analysis of the expression patterns of genes that are the targets of miRNAs and analysis of the transcriptome at the maize V3 (third leaf) stage.

## Materials and methods

### Plant materials and inoculation with RBSDV

Seeds of B73, a highly susceptible maize inbred line, were sown in pots in a field in the natural environment of Nanjing, Jiangsu Province, China. Two weeks later, each seedling at the third leaf (V3) stage was exposed for 3 d in inoculation chambers to 100 small brown planthoppers (SBPH, *Laodelphax striatellus*) that were 1% viruliferous for RBSDV. The SBPH were removed from leaves on the third day. At the same time, other B73 maize seedlings at the V3 stage were exposed to virus-free SBPH as controls. Viruliferous SBPH for inoculation were collected from rice fields in Nanjing, Jiangsu Province, China. The virus-free SBPH were propagated for many generations by feeding them on plants that were not infected with RBSDV or RSV. Untreated maize plants were grown under the same conditions as control plants. There were three technical replicates for each of the three treatments: viruliferous SBPH, virus-free SBPH, and untreated. Leaves were collected at 0 d (control, B0), 1.5 d (viruliferous, BP1.5; virus-free, BN1.5), and 3 d (viruliferous, BP3; virus-free, BN3) after inoculation. Leaves for qRT-PCR verification in three pathways were collected at V3a (1.5 d), V3b (3 d), V6 (six-leaf stage), V9 (nine-leaf stage) and V12 (12-leaf stage) after inoculation with viruliferous or virus-free SBPH. There were three biological replicates for each of the sequencing samples B0, BN1.5, BP1.5, BN3, and BP3 in the transcriptome sequencing, and the correlation (*R* ^2^) between the three biological replicates was calculated. qRT-PCR labeling of a TaqMan® probe specific for RBSDV-S2 (Supplementary Table S1 at *JXB* online) was used to confirm successful infection at 1.5, 3, 6, and 20 d after inoculation (DAI). Three technical replicates were performed for detection of viral infection. When all the seedlings had reached the V4 (fourth leaf) stage, they were transplanted to the field and grown inside insect-proof netting. By 1 month after transplanting, all the plants had reached the VT (tasseling) stage, and the rates of infection were determined (= number of plants with MRDD symptoms/number of plants inoculated). Leaves with or without MRDD symptoms were then collected to investigate the characteristics of the virus particles and cytological variation among plants by transmission electron microscopy (H-7500, HITACHI, Japan). Images were recorded using a Gatan 832 CCD camera (Gatan Inc., Pleasanton, CA, USA).

### RNA extraction, library construction and sequencing

For every RNA-seq library, five plants were combined per sample. Total RNA was extracted using TRIzol® Reagent (Invitrogen, CA, USA) following the manufacturer’s procedure. Total RNA quantity and purity were analyzed using a Bioanalyzer 2100 and RNA 6000 Nano LabChip Kit (Agilent, CA, USA) with RIN number >7.0. Approximately 1 μg of total RNA was used to prepare a small RNA library according to the TruSeq Small RNA Sample Prep Kit protocol (Illumina, San Diego, USA). Single-end sequencing (36bp) was then performed on an Illumina HiSeq2500 at the facilities of LC-BIO (Hangzhou, China), following the manufacturer’s recommended protocol.

Approximately 20 μg of total RNA were used to prepare the degradome library. First, poly (A)^+^ RNA was used as input RNA and annealed with biotinylated random primers. These biotin-tagged RNA fragments were then captured on streptavidin. Those RNAs containing 5′-monophosphates were then ligated with a 5′ adaptor. First-strand cDNA was then synthesized and the PCR product was amplified. Libraries were sequenced using the 5′ adapter only, resulting in the sequencing of the first 36 nucleotides of the inserts that represented the 5′ ends of the original RNAs. We then performed single-end sequencing (36bp) on an Illumina HiSeq2500 at the facilities of LC-BIO (Hangzhou, China) following the manufacturer’s recommended protocol.

Approximately 15 μg of total RNA from leaves that had been inoculated with either viruliferous or RBSDV-free SBPH were used to prepare the transcriptome library. Poly-A containing RNA was isolated from total RNA using Oligo (dT) magnetic beads. The mRNAs were broken into fragments in fragmentation buffer. First-strand cDNA was then synthesized using random hexamers with the mRNA template. The second-strand was synthesized in a reaction mixture including buffer, dNTPs, Rnase H, and DNA polymerase I. Fragments were purified using the Qiaquick® kit and eluted with EB buffer (10mM Tris·Cl, pH 8.5), and then termini were repaired. The fragments were size-selected using agarose gel electrophoresis, and then amplified by PCR. Sequencing of the constructed library was then performed. The raw data have been uploaded into the NCBI database SRA (PRJNA299369). The SRR numbers for transcriptome sequencings were from SRR2758148 to SRR2758177.

Two small RNA libraries, one degradome library, and fifteen RNA-Seq libraries were constructed from maize infected with either viruliferous or virus-free SBPH. Fifteen RNA-Seq libraries were constructed from the three biological replicates of the leaf tissue samples B0, BN1.5, BP1.5, BN3, and BP3. Two small RNA libraries were constructed from the mixed RNAs obtained from the three technical replicates at 3 DAI with viruliferous or virus-free SBPH. The degradome library was also constructed from mixed RNAs obtained from the three technical replicates sampled at 3 DAI with viruliferous or virus-free SBPH. The SRR numbers for the miRNA and degradome sequencings were SRR2763387, SRR2763388, and SRR2763867.

### Sequence data analysis

Adapters and low-quality reads were filtered to obtain raw sequence reads. Unique sequences 18–26 nucleotides in length were mapped to specific species’ (such as *Medicago truncatula* or *Vitis vinifera*) precursors in miRBase 20.0 using blast searches to identify known miRNAs and novel 3p- and 5p-derived miRNAs. The unique sequences that mapped to a hairpin arm of a specific species of mature miRNA were identified as known miRNAs. The unique sequences mapping to the other arm of a known specific species precursor hairpin opposite the annotated mature miRNA-containing arm were considered to be novel 5p- or 3p-derived miRNA candidates. The remaining sequences were mapped to other selected species precursors (with the exclusion of specific species) in miRBase 20.0 by blast, and the mapped pre-miRNAs were used as further blast queries against the specific species genomes to determine their genomic locations. Thus, miRNAs in the above two categories were defined as known miRNAs.

For digital sequencing data, the adaptors, empty tags (no tag sequence between the adaptors), low-quality tags (tags containing one or more unknown nucleotides ‘N’), and tags with a copy number equal to one were removed from the raw data to obtain 50-bp clean tags. All clean tags were mapped to the assembled transcriptome data generated by RNA-Seq. The number of tags corresponding to each gene was calculated and normalized to the RPKM (the number of transcripts per million clean tags per kilobase) to analyze the expression of DEGs. The significance threshold was set at 0.05 to identify genes expressed differentially at individual time points.

*De novo* transcriptome was assembled from transcriptome sequence data. More than 4G of data were assembled using Trinity (Bankar *et al.*, 2015). Sequence homologies for the assembled Trinity contigs were identified by local blastall against sequences in the NCBI non-redundant (nr) protein database and the Swiss-Prot database (E-value <1e-10). Genes were thus tentatively identified according to the best hits against known sequences. In the present study, miRNAs and genes with log_2_FC (fold-change) greater than one and *P*-value less than 0.01 were considered to have altered expression.

### Verification by qRT-PCR

The validity of miRNA sequences and RNA-Seq were verified by quantitative real-time PCR (qRT-PCR). Two miRNAs (miR169i-p5 and miR8155), two target genes (GRMZM2G069316 and GRMZM2G031169), and eight DEGs (up-regulated: GRMZM2G031790, GRMZM2G113191, GRMZM2G070620, and GRMZM2G029912; down-regulated: GRMZM2G120619, GRMZM2G057281, GRMZM2G155216, and GRMZM2G101664) with differential expression at 1.5 and 3 DAI were validated using qRT-PCR. First-strand cDNAs of miRNAs and genes were synthesized using a miRcute miRNA First-Strand cDNA Synthesis Kit (KR201) or Fast Quant RT Kit (KR106) (TIANGEN, China). The qRT-PCR reaction was performed using Bio-Rad iQ5 following the instructions for the miRcute miRNA qRT-PCR Detection Kit (SYBR Green) or SuperReal PreMix Plus (SYBR Green) (FP205). Three biological replicates were sampled and three technical replicates of all reactions were performed on each sample. The 5S rRNA (Lang *et al.*, 2011) and *ZmTubulin* (Zuo *et al.*, 2015) genes were used as internal controls for miRNA and transcription level quantitation. The expression from growth stages V3 to V12 of miR8155 and target gene GRMZM2G031169, and DEGs relating to the metabolic pathways zma01100 and zma01110 in the Kyoto Encyclopedia of Genes and Genomes (KEGG) database were also verified by qRT-PCR. All primers are listed in Supplementary Table S13.

## Results

### Inoculation of maize with RBSDV

The inoculation status of plants with RBSDV was confirmed by qRT-PCR using RBSDV-S2 probe primers at 1.5, 3, 6, and 20 DAI. RBSDV could be detected in maize plants that had been inoculated at the V3 stage with viruliferous SBPH, but not in the plants exposed to virus-free SBPH. Virus content increased gradually as the maize plants grew (1.5, 3, 6, and 20 DAI) (Supplementary Fig. S1a, b). MRDD symptoms such as stunting, dark-green leaves and sheaths that appear rough due to waxy enations, or failure of heading at 1 month after transplanting were evident on maize plants that had been infected with viruliferous SBPH (Supplement Fig. S1c). There were no diseased plants in the non-inoculated group and no virus-free plants in the SBPH-infected group (Supplement Fig. S1c). RBSDV particles with a diameter of 70–80nm were observed in the cytoplasm of diseased leaves (Supplement Fig. S1d). In addition, crystallized virus particles were detected in the cytoplasm of diseased leaves at the VT growth stage (Supplement Fig. S1e).

### Response of known miRNAs to RBSDV in maize

**Fig. 1.**
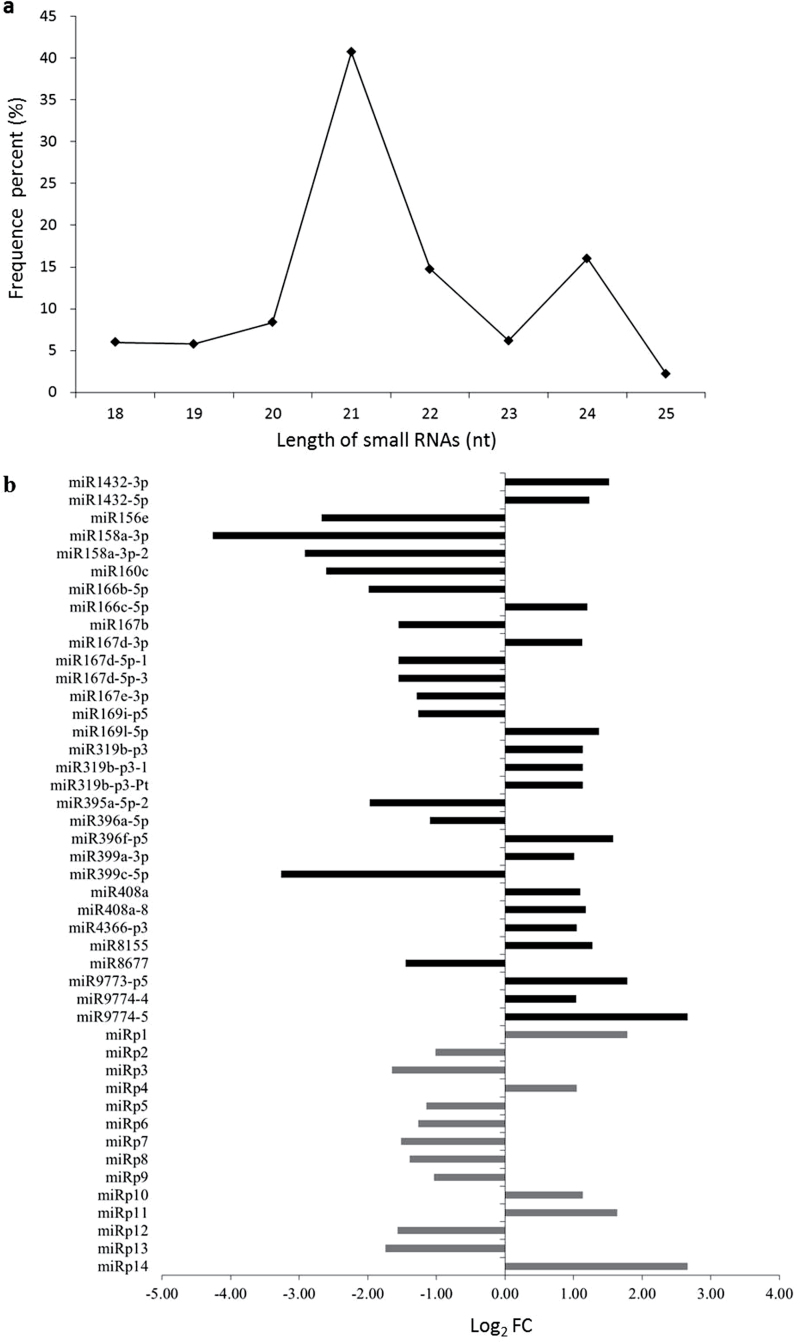
Summary of miRNA sizes and differentially expressed miRNAs. (a) The size distribution of unique miRNAs. (b) Differential expression of miRNAs in RBSDV-infected maize. FC, fold change. Black bars represent known miRNAs; grey bars represent unknown miRNAs.

A total of 13175409 and 11065903 raw reads were generated from libraries prepared from maize leaf tissue that had been grown after inoculation with BN3 (virus-free) or BP3 (viruliferous) vectors, respectively, and 2615920 and 1888049 raw unique reads were generated by deep sequencing miRNAs from BN3 and BP3, respectively. Among the unique miRNAs, sequences 21 and 24 nt in length were predominant, and 21-nt sequences were the most abundant in these two libraries (Fig. 1a).

Unique sequences of 18–25 nucleotides in length were mapped to species precursors in miRBase 20.0 (http://microrna.sanger.ac.uk/) by blast to identify known miRNAs and novel miRNA sequences derived from 3′ (3p in miRNA names) and 5′ (5p in miRNA names) ends. In the two small RNA libraries from plants cultivated with viruliferous or virus-free SBPH, 525 miRNAs representing 123 known miRNA families and 299 novel miRNAs were identified. The known and novel miRNAs from the viruliferous and virus-free libraries with at least two-fold change in expression (Log_2_ FC≥1) and at least 10 reads in a dataset are shown in Supplementary Table S2. Three non-conserved and 28 conserved miRNAs representing 17 known miRNA families were identified (Supplementary Table S3). These 31 known miRNAs were detected on chromosomes 1–9, and on the chloroplast and mitochondrial genomes. The lengths of these miRNA precursors varied from 25 to 334 nt (Supplementary Table S3). Three non-conserved miRNAs (miR160c, miR167d-5p-1, and miR167d-5p-3) were expressed at least three-fold lower in the viruliferous maize than in the virus-free maize. Among the conserved miRNAs, members of eight families were highly abundant, while members of five families were expressed at low levels. The expression patterns of miRNAs from the miR166, miR167, miR169, and miR399 families were not completely uniform. The miR166b-5p, miR167b, miR167d-5p-1, miR167d-5p-3, miR167e-3p, miR169i-p5, and miR399c-5p miRNAs were expressed at low levels, while miR166c-5p, miR167d-3p, miR169l-5p, and miR399a-3p were highly expressed (Supplementary Table S3).

### Novel miRNAs in response to infection with RBSDV

Compared with the group of known miRNAs identified in the library prepared from virus-free plants, 299 novel miRNA sequences were identified in the library prepared from virus-infected plants. Among these, 156 novel miRNAs were derived from the 3′ ends of the pre-miRNAs, and the rest were derived from the 5′ ends. Only 14 novel miRNAs (named miRp1-14) showed at least a two-fold change in expression and at least 10 reads per dataset (Supplementary Table S4). These 14 novel miRNAs were mapped to all maize chromosomes, except for chromosome 9. The lengths of these 14 novel unique miRNA sequences were either 20, 21, or 24 nt, and the majority were 21 and 24 nt in length. The lengths of these 14 novel unique miRNA precursors ranged from 59 to 420 nt (Supplementary Table S4). Five novel miRNAs (miRp1, miRp4, miRp10, miRp11, and miRp14) were highly expressed with norm and raw values in two libraries all greater than 10, and nine novel miRNAs (miRp2, miRp3, miRp5, miRp6, miRp7, miRp8, miRp9, miRp12, and miRp13) were expressed at low levels with norm and raw values in two libraries lower than 10 (Supplementary Table S4).

### miRNA target genes

**Fig. 2.**
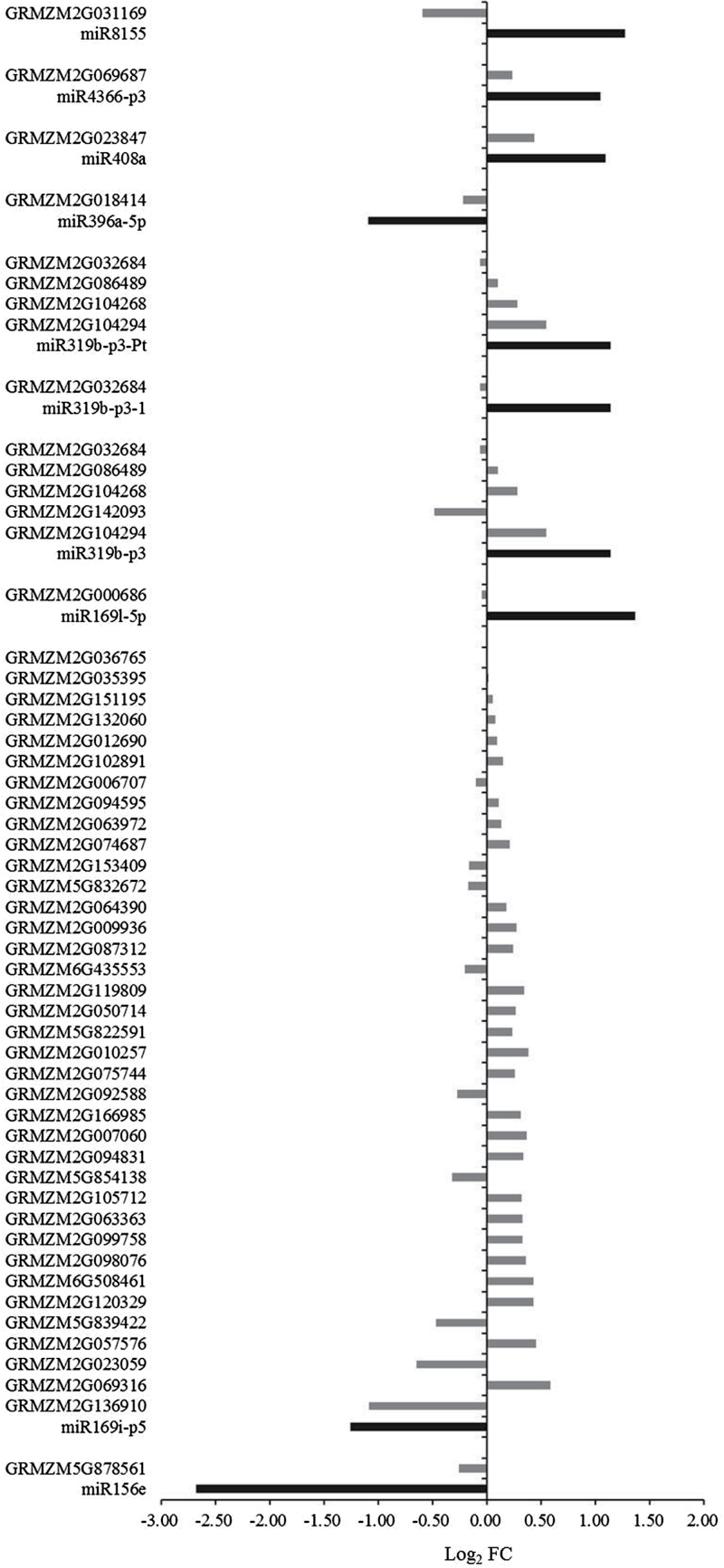
Differential expression of the known miRNAs and target genes in virus-inoculated or virus-free maize. FC, fold change. Black bars represent expression of miRNAs; grey bars represent expression of target genes.

To identify the target genes of the known and novel miRNAs identified here, prediction of miRNA targets and sequencing of the degradome were performed. A total of 10^7^ raw reads and 4.05×10^6^ unique raw reads were generated from the viruliferous library for analysis of the target genes for miRNAs. The software CleaveLane 3.0 was used to identify known and novel miRNAs in the data generated for target genes (Addo-Quaye *et al.*, 2009). A total of 3945 transcripts (prediction and sequencing) from 2823 genes for 633 miRNAs were predicted to be microRNA target genes. A total of 688 transcripts from 502 genes for 37 miRNAs were identified in the degradome library (Supplementary Table S2). A total of 99 transcripts from 48 genes were identified for 10 known miRNAs (miR156e, miR169i-p5, miR169l-5p, miR319b-p3, miR319b-p3-1, miR319b-p3-Pt, miR396a-5p, miR408a, miR4366-p3, and miR8155) that exhibited at least two-fold change in expression and that had at least 10 reads per dataset. Among these miRNAs, seven (miR169l-5p, miR319b-p3, miR319b-p3-1, miR319b-p3-Pt, miR408a, miR4366-p3, and miR8155) were up-regulated. Nine genes targeted by these seven miRNAs (Fig. 2) included a P450 reductase, an oxidoreductase family protein, and an NAD(P)-binding Rossmann-fold superfamily protein (Supplementary Table S5). Three other miRNAs (miR156e, miR169i-p5, and miR396a-5p) were down-regulated (Fig. 2). Only one target gene each was identified for miR156e and miR396a-5p, namely a *SQUAMOSA* promoter-binding (SPB) protein and a growth-regulating factor, respectively (Supplementary Table S5). For miR169i-p5, 37 target genes were detected, including a 60S acidic ribosomal protein family, an abscisic stress-ripening protein, a serine/threonine-protein kinase Rio1, and a ubiquitin-related protein (Supplementary Table S5).

**Fig. 3.**
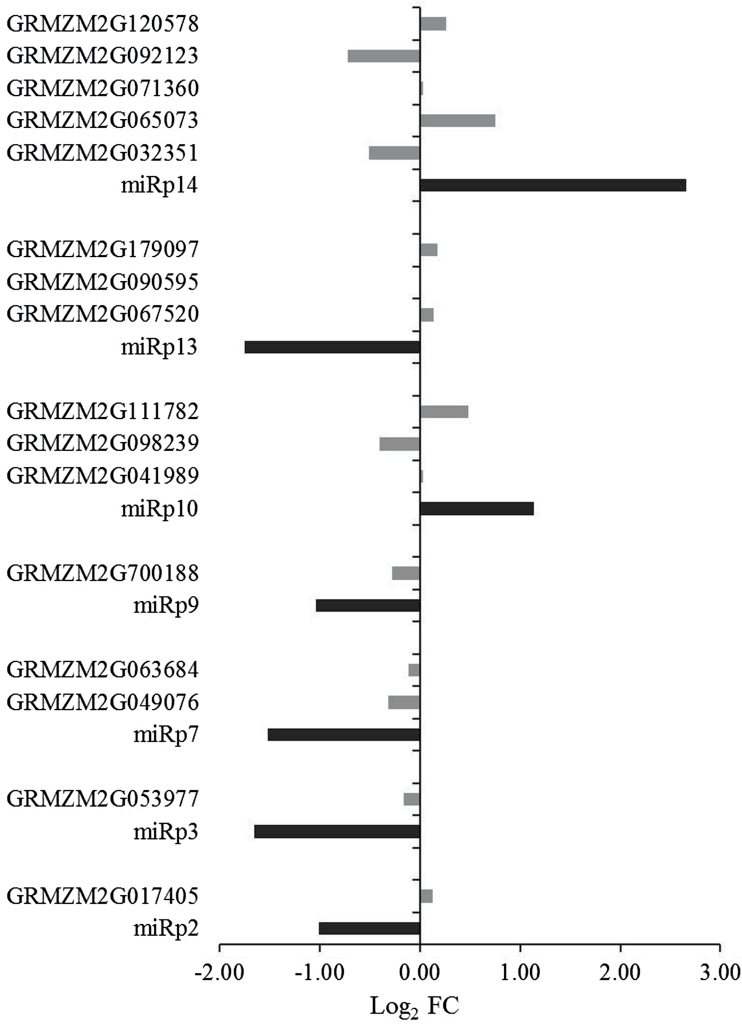
Differential expression of novel miRNAs and target genes in virus-infected and virus-free maize. FC, fold change. Black bars represent expression of miRNAs; grey bars represent expression of target genes.

A total of 32 transcripts from 29 target genes for seven novel miRNAs were predicted (Supplementary Table S6). These target genes were related to nucleic acid binding, transferase activity, zinc ion binding, and oxidation–reduction functions, among others. However, target genes for novel miRNAs were not identified in the degradome library. The expression levels of 16 predicted target genes for seven miRNAs were affected by infection with RBSDV according to RNA-Seq results, and their targets included a protein kinase superfamily protein and phototropin (Fig. 3, Supplementary Table S6). The expression of five of these target genes changed significantly (*P*<0.05), and three of the target genes for two of the miRNAs were negatively regulated. GRMZM2G092123, a target gene for miRp14, is annotated as a tetratricopeptide repeat (TPR)-like superfamily member and was down-regulated by 1.67-fold (*P*=3.44×10^–4^).

### The RBSDV-responsive transcriptome in maize

**Fig. 4.**
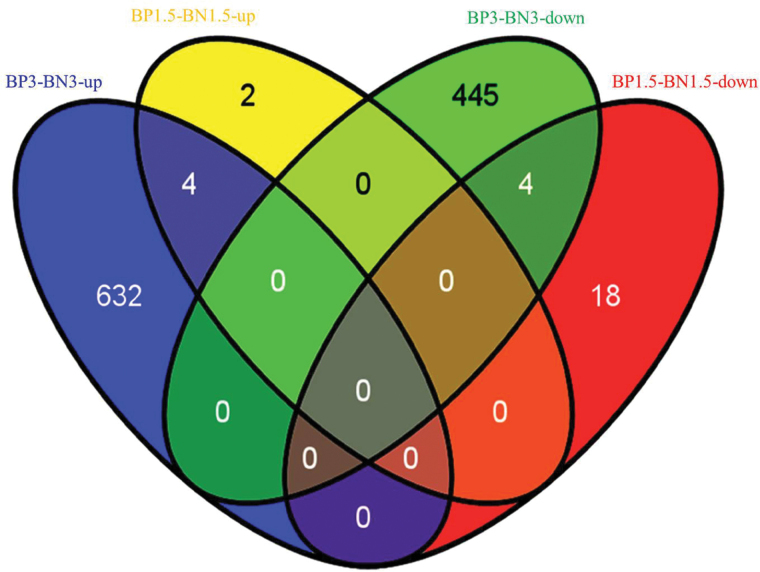
Relationships of changes in gene expression based on transcriptome sequencing. BP3-BN3-up, up-regulated genes at 3 DAI of maize with viruliferous small brown planthopper (SBPH ) compared with inoculation with virus-free SBPH. BP3-BN3-down, down-regulated genes at 3 DAI of maize with viruliferous SBPH compared with inoculation with virus-free SBPH. BP1.5-BN1.5-up: up-regulated genes at 1.5 DAI of maize with viruliferous SBPH compared with inoculation with virus-free SBPH. BP3-BN3-down: down-regulated genes at 1.5 DAI of maize with viruliferous SBPH compared with inoculation with virus-free SBPH. (This figure is available in colour at JXB online).

The numbers of differentially expressed genes (DEGs) identified by transcriptome sequencing are shown in Table 1. A total of 28 and 1085 DEGs were detected in the BP1.5–BN1.5 and BP3–BN3 libraries, respectively, of which four up-regulated DEGs (3-ketoacyl-CoA synthase, a bifunctional monodehydroascorbate reductase and carbonic anhydrasenectarin-3 precursor, a cytochrome P450 protein, and *WAX2*) and four down-regulated DEGs (three chlorophyll A-B binding proteins and one zinc finger family protein) were detected in both BP1.5–BN1.5 and BP3–BN3 (Fig. 4, Supplementary Table S7). The up-regulated DEGs were related to functions including iron ion binding and fatty acid biosynthesis. The expression of GRMZM2G070620, which was annotated as a cytochrome P450 protein, changed most significantly with log_2_FC values of 6.0191 and 2.2686 in BP1.5–BN1.5 and BP3–BN3, respectively. Three of four down-regulated DEGs were annotated as chlorophyll A–B binding protein, and the other was annotated as a zinc finger family protein (Supplementary Table S7).

**Table 1.** Summary of analyses of transcriptome sequences from individual libraries

Gene classification	Change in expression	BP–BN		BP–B0		BN–B0	
		1.5 d	3 d	1.5 d	3 d	1.5 d	3 d
Total DEGs	increased	11108	10499	9933	10338	10856	10933
	decreased	10090	9571	9811	9921	10233	10114
	total	21198	20070	19744	20259	21089	21047
Significant DEGs	increased	6	636	3166	1937	3607	1446
	decreased	22	449	3566	2832	3837	3499
	total	28	1085	6732	4769	7444	4945

BP: inoculated with viruliferous small brown plant hopper (SBPH); BN: inoculated with virus-free SBPH; B0: day zero control; Significant DEGs: |log_2_FC| > 1; *P*<0.01

Dwarfing is one of the typical symptoms of MRDD; accordingly, the expression of genes related to cell wall development, such as cellulose synthase and pectinesterase, lignin biosynthesis, and those related to plant stature, such as gibberellin and auxin biosynthetic enzymes, was affected in virus-infected plants. In BP3–BN3, the expression of DEGs for pectinesterase, protein kinase, a probable mannan synthase, *CSLA*1 (GRMZM2G105631), and a chitinase family protein precursor was increased. The expression of cellulose synthases, including *CSLF* 6 (GRMZM2G122277, GRMZM2G110145) and *CESA* 3 (GRMZM2G025231), declined significantly (Supplementary Table S8). In virus-infected plants, the expression of pectinesterase (GRMZM2G019411) and a high-light inducible protein (GRMZM2G019807) also decreased significantly (*P*<0.01) with log_2_FC values relative to uninfected plants of –1.1461 and –1.0275, respectively. The expression of two lignin-related genes (GRMZM2G169033 and GRMZM2G320786) also increased significantly (*P*<0.01) with log_2_FC values relative to uninfected plants of 3.2525 and 2.2729, respectively. In BP1.5–BN1.5, the expression of only one glycosyl hydrolase gene (GRMZM2G060837) related to cell wall function changed significantly (log_2_FC value −1.3368, *P*=1.83×10^−05^). These results showed that the behavior of cell walls and the expression of genes related to the function of cell walls were both influenced by RBSDV infection. The decrease in the expression of cellulose synthase might be one reason for the dwarfing symptoms observed in infected plants. The expression of five gibberellin-related genes and 12 auxin-related genes was also altered significantly in BP3–BN3 (*P*<0.01) (Supplementary Table S8). The functions of gibberellin-related genes with altered expression included a gibberellin receptor GID1L2, gibberellin 2-beta-dioxygenase, and gibberellin 20-oxidase 2. Auxin-related genes with altered expression included the auxin-induced protein 5NG4, an auxin efflux carrier component, an auxin-repressed protein, homologs of the rice auxin-responsive Aux/IAA gene family member OsIAA, and an auxin-responsive SAUR gene family member, OsSAUR.

Typically, plant leaves suffering from MRDD are dark green and appear rough due to waxy enations (Tao *et al.*, 2013*a*,b). The leaf symptoms could be due to photosystem damage or to changes in the chloroplast. In our study, among 27 chloroplast-related DEGs, eight were up-regulated, and 19 were down-regulated (Supplementary Table S6), of which three (GRMZM2G024150, GRMZM2G005433, and AC190623.3_FG001) are related to the photosystem. Because photosynthesis takes place in chloroplasts, these organelles contain chlorophyll, carotenoids, and cytochromes. In BP3–BN3 and BP1.5–BN1.5, 14 photosystem-related genes showed significantly suppressed expression. Among these, one gene (GRMZM2G057281) was significantly suppressed in both BP3–BN3 and BP1.5–BN1.5, but the other 13 of these genes were only detected in BP3–BN3. The expression of genes related to the functions of chlorophyll, carotenoids, and cytochromes showed significant alterations in BP3–BN3 and BP1.5–BN1.5. The expression of three chlorophyll A–B binding proteins (GRMZM2G120619, GRMZM2G057281, and GRMZM2G155216) was suppressed in BP3–BN3 and BP1.5–BN1.5, and that of other chlorophyll-related genes was only detected in BP3–BN3. Except for the expression of chlorophyllase-2 (GRMZM2G170734) with a fold increase of 4.8974, the expression of all of the chlorophyll-related and carotenoid-related genes decreased. However, the expression of cytochrome-related genes was also significantly affected. The above results indicated that chloroplasts and photosynthesis were affected by RBSDV infection through the down-regulation of genes for chlorophyll A–B binding protein (Supplementary Table S9).

The expression of genes related to plant hormone biosynthesis, disease defense, or plant stress responses also often change significantly under biotic stresses. In the present study, the expression of three pathogenesis-related genes (GRMZM2G072612, GRMZM2G028928, and GRMZM2G108537), which correspond to pathogenesis Gene Ontology (GO) terms GO:0009405 and GO:0009406, increased in BP3–BN3 (Supplementary Table S10). Several identified DEGs were related to disease defense and stress resistance functions, including stress resistance signal transduction, and transcriptional regulation. The resistance-related genes detected in the significant DEG dataset included seven glutathione S-transferase genes, nine peroxidase genes, five heat shock protein genes, two ferredoxin-nitrite reductase genes and five chitinase genes (Supplementary Table S10). DEGs related to signal transduction, such as an LRR disease-resistance protein and protein kinases including a lectin protein kinase, a receptor kinase, a serine/threonine-protein kinase, and a tyrosine protein kinase, were also detected. The expression of pathogenesis-related transcription factors was also altered, and these included basic helix-loop-helix, ethylene-responsive (ERF114), GRAS family, MYB family, heat stress, HBP-1b, and WRKY family genes.

Protein degradation pathways such as the ubiquitin proteasome system participate in multiple biotic and abiotic stresses. Our results corroborated the hypothesis that the ubiquitin proteasome system might be part of the response to RBSDV infection. Through transcriptome analysis, three ubiquitin biosynthesis-related genes (GRMZM2G046848, GRMZM2G144782, and GRMZM2G303964) with significantly altered expression were detected in BP3–BN3 (Table 2). The expression of two of these genes (GRMZM2G046848 and GRMZM2G144782) increased, with log_2_FC values of 1.3561 and 1.0710, respectively, while that of GRMZM2G303964 declined, with a log_2_FC value of −1.7203. Each of these genes correspond to ubiquitin GO terms (GO:0000151, GO:0004842, and GO:0016567, respectively) or to the ubiquitin pathway (zma04120) in the KEGG database. This result suggests that ubiquitin-related genes respond to RBSDV infection in maize (Table 2).

**Table 2.** Details regarding changes in expression of ubiquitin-related genes in maize infected with RBSDV

Gene	Log_2_ FC	*P*-value	Description	GO	KEGG
GRMZM2G046848	1.3561	4.39E-03	protein kinase	GO:0000151//ubiquitin ligase complex	
				GO:0004672//protein kinase activity	
				GO:0004674//protein serine/threonine kinase activity	
				GO:0004842//ubiquitin-protein ligase activity	
				GO:0005524//ATP binding	
				GO:0006468//protein phosphorylation	
				GO:0016567//protein ubiquitination	
GRMZM2G144782	1.0710	8.92E-05	RING finger and CHY zinc finger domain-containing protein 1	GO:0005515//protein binding	zma:100192978 zma04120//ubiquitin mediated proteolysis
				GO:0008270//zinc ion binding	
				GO:0005515//protein binding	
GRMZM2G303964	−1.7203	4.42E-05	U-box domain containing protein	GO:0000151//ubiquitin ligase complex	
				GO:0004842//ubiquitin-protein ligase activity	
				GO:0005488//binding	
				GO:0016567//protein ubiquitination	
				GO:0042309//homoiothermy	
				GO:0050825//ice binding	
				GO:0050826//response to freezing	

**Fig. 5.**
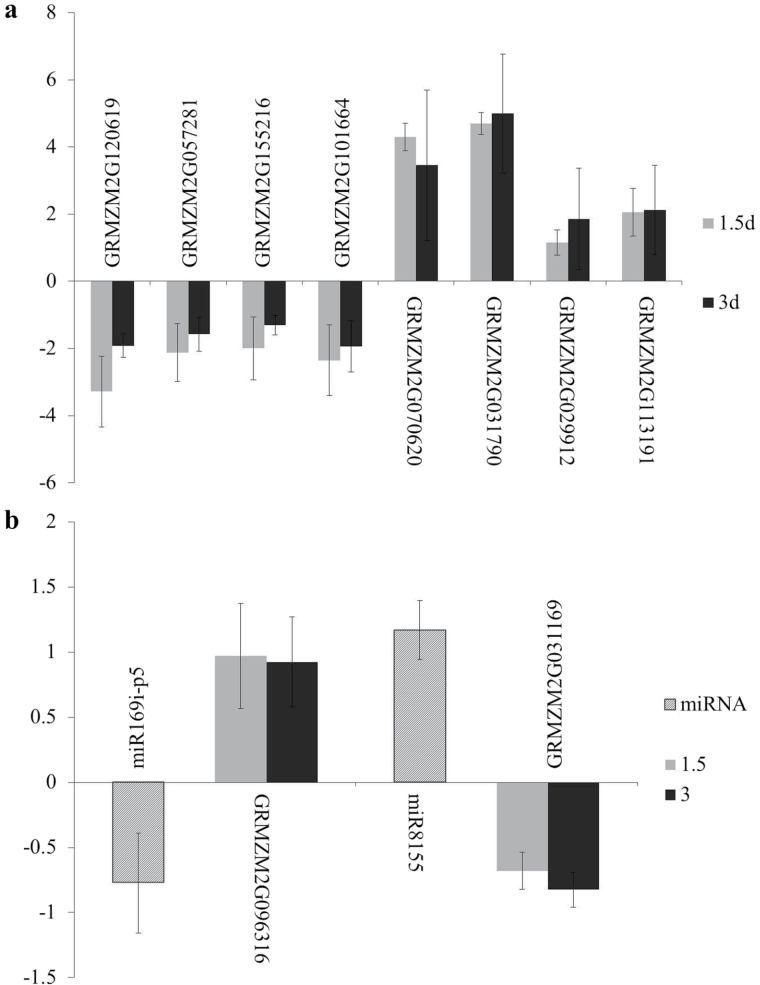
Confirmation by qRT-PCR of (a) differentially expressed genes (DEGs), and (b) miRNAs and target genes identified in sequence data. *ZmTubulin* and *5S rRNA* were used as stably expressed reference genes. The expression levels of each mRNA and miRNA were normalized by comparison with their expression in virus-free maize. The experiments were repeated with three biological and three technical replicates; bars indicate the standard error of the biological replication.

To confirm the RNA-Seq results for the RBSDV-responsive genes at different time points, the expression patterns of eight DEGs (GRMZM2G120619, GRMZM2G057281, GRMZM2G155216, GRMZM2G101664, GRMZM2G 031790, GRMZM2G113191, GRMZM2G070620, and GRMZM2G029912) were validated by qRT-PCR in RNA samples from leaves harvested at both 1.5 and 3 DAI. As the results shown in Fig. 5a indicate, GRMZM2G120619, GRMZM2G057281, GRMZM2G155216, and GRMZM2G 101664 were down-regulated at both 1.5 and 3 DAI. The expression of GRMZM2G031790, GRMZM2G113191, GRMZM2G070620, and GRMZM2G029912 increased at 1.5 d and 3 DAI with RBSDV.

### Negatively regulated miRNAs target genes

**Fig. 6.**
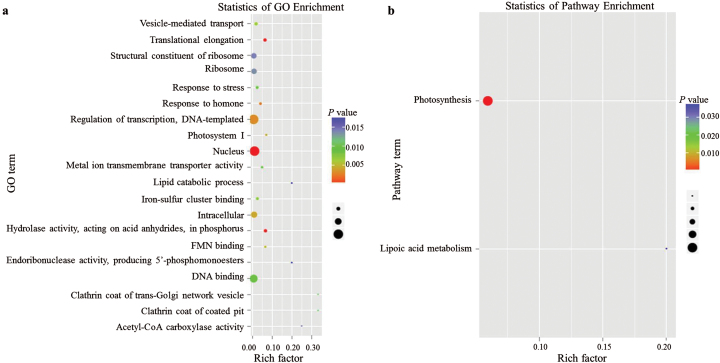
Analysis of the targets of identified miRNAs in maize that are responsive to infection by RBSDV. (a) Profile of GO analysis of the targets of identified responsive miRNAs in maize infected with RBSDV. (b) Profile of KEGG analysis of the targets of identified responsive miRNAs in maize infected with RBSDV. (This figure is available in colour at JXB online).

**Fig. 7.**
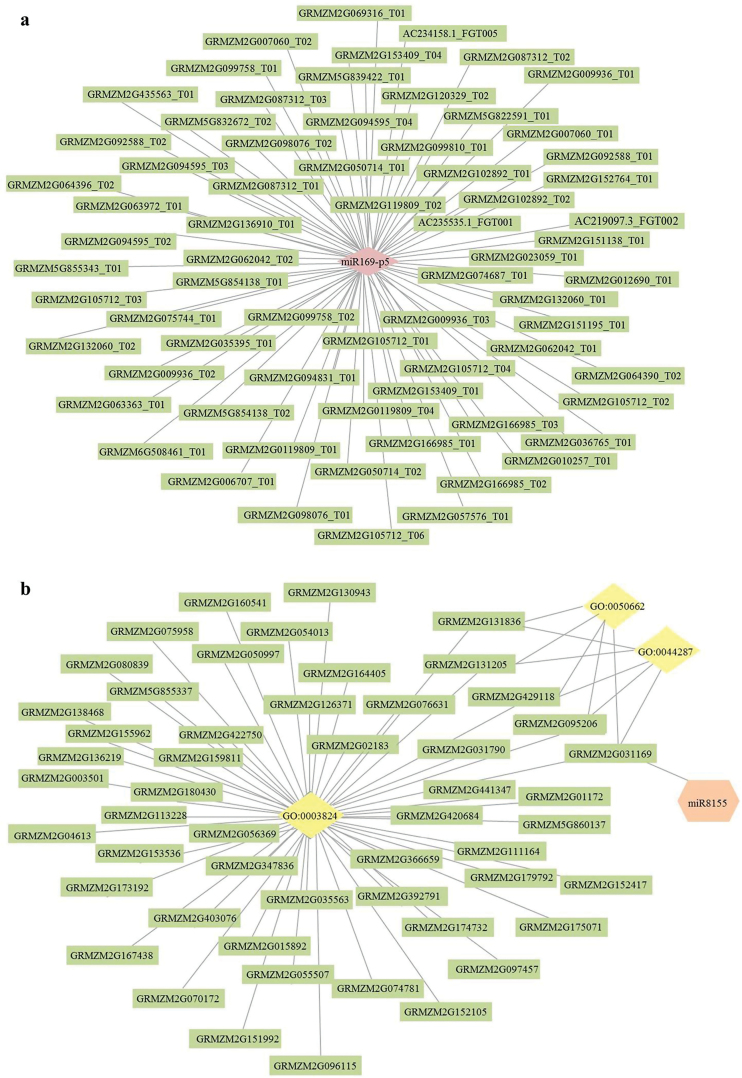
Networks of miR169i-p5 and miR8155 associated with GO terms of target genes. (a) Network of miR169i-p5-GRMZM2G069316 associated with one GO term (GO:0005488//binding). (b) Network of miR8155-GRMZM2G031169 associated with three GO terms (GO:0003824//catalytic activity; GO:0044237//cellular metabolic process; GO:0050662//coenzyme binding). (This figure is available in colour at JXB online).

Analysis of these miRNA degradomes and transcriptomes indicated that most genes with altered expression were enriched in GO terms related to the nucleus, regulation of transcription and DNA templates, and DNA binding (Fig. 6a). Genes related to photosynthesis in KEGG were also enriched (Fig. 6b). To detect negatively regulated miRNAs and their target genes, combined analysis of the expression of miRNAs, the degradome, and the transcriptome was performed. A total of 30 negatively regulated genes, which are targeted by six miRNAs (miR169i-p5, miR169l-5p, miR319b-p3, miR319b-p3-1, miR319b-p3-Pt, and miR8155), were identified (Supplementary Table S5). Among these 30 genes, the expression of only five (GRMZM2G069316, GRMZM2G057576, GRMZM2G120329, GRMZM2G142093, and GRMZM2G031169) changed significantly (*P*<0.05). Furthermore, the expression of two target genes GRMZM2G069316 (targeted by miR169i-p5) and GRMZM2G031169 (targeted by miR8155) changed more than 1.5-fold (|log_2_FC| > 0.58; *P*<0.05) (Supplementary Table S5). GRMZM2G069316 was related to only one GO term (GO:0005488//binding) (Fig. 7a). Thirteen DEGs were detected for GO:0005488, which included annotations for HEAT repeat family proteins, a lectin-like protein kinase, a mitochondrial carrier protein, and a U-box domain-containing protein component of ubiquitin ligase (Supplementary Table S11). GRMZM2G031169 was related to three GO terms (GO:0003824//catalytic activity; GO: 0044237//cellular metabolic process; and GO:0050662//coenzyme binding) (Fig. 7b). Fifty-one DEGs that were detected were related to GO:0003824, and were annotated as a 3-ketoacyl-CoA synthase, an alpha-amylase precursor, an AMP-binding domain-containing protein, a protein phosphatase 2C, a reticuline oxidase-like protein precursor, and UDP-glucuronate 4-epimerase (Supplementary Table S12). The same four DEGs were detected in each of GO:0003824, GO:0044237, and GO:0050662, which were annotated as a dehydrogenase/reductase SDR family member 12, a dehydrogenase, and UDP-glucuronate 4-epimerase, respectively (Supplementary Table S12).

To confirm the miRNA and transcriptome sequencing results for the maize response to RBSDV, qRT-PCR was used to validate two miRNAs, miR169i-p5 and miR8155, and their two target genes, GRMZM2G069316 and GRMZM2G031169, respectively. As shown in Fig. 5b, the expression of miR169i-p5 decreased, but that of miR8155 increased. Expression of GRMZM2G069316, which is the target gene for miR169i-p5, increased both at 1.5 and 3 DAI with RBSDV. The target gene for miR8155 (GRMZM2G031169) was down-regulated at both 1.5 and 3 DAI. These results were consistent with the results of analysis of sequence data for miRNAs and the transcriptome.

### Metabolic pathway analysis at five maize growth stages

**Fig. 8.**
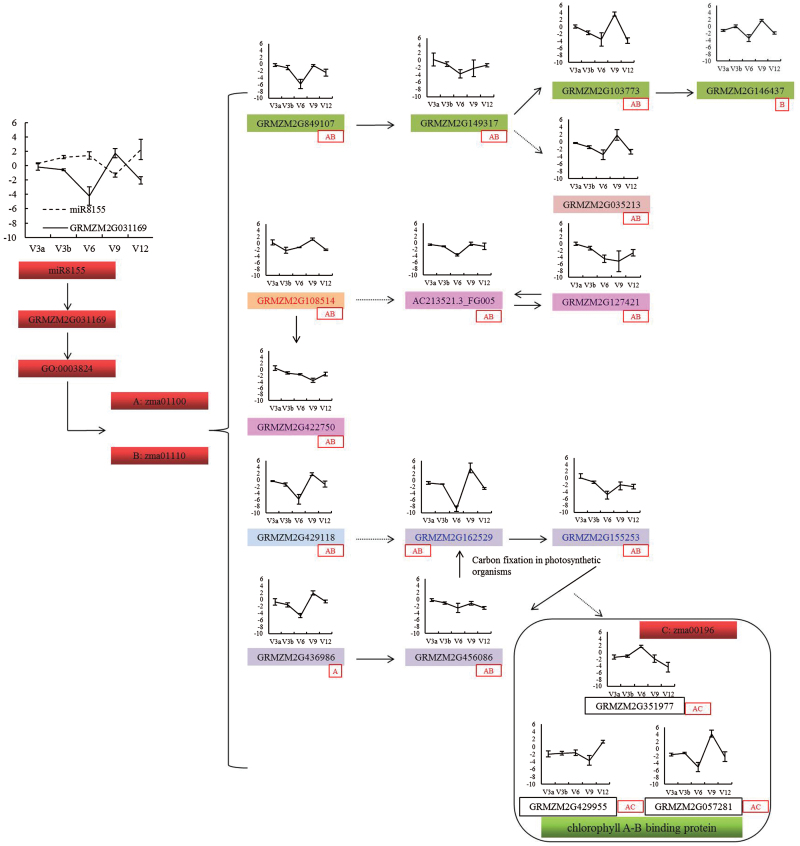
Analysis of gene expression in three pathways from growth stages V3 to V12 of RBSDV-infected maize. Genes with the same background color indicates that they are in the same metabolism group. Genes GRMZM2G162529 and GRMZM2G155253 are written in a different color to represent the fact that they are in he two metabolism groups. Solid lines represent actual relationship in the metabolism pathways; dotted lines represent possible relationships in the metabolism pathways. ‘A’ in a red box indicates that the gene takes part in zma01100; ‘B’ in a red box indicates that the gene takes part in zma01110; ‘A’ and ‘B’ in a red box indicates that the gene simultaneously takes part in zma01100 and zma01110; ‘A’ and ‘C’ in a red box indicates that the gene simultaneously takes part in zma01100 and zma00196. V3a represents maize at 1.5 d after inoculation (DAI); V3b represents 3 DAI. (This figure is available in colour at JXB online).

Based on miRNA and DEGs, the expression of genes involved in KEGG metabolic pathways zma01100 and biosynthesis of secondary metabolites (zma01110) were investigated at the V3, V6, V9, and V12 maize growth stages. miR8155 was up-regulated at V3, V6, and V12, but down-regulated at V9. As the target gene of miR8155, GRMZM2G031169 was correspondingly regulated at these growth stages.The gene ontology term GO:0003824, which includes GRMZM2G031169 and 51 other DEGs, mainly related to the two pathways zma01100 and zma01110. Six DEGs are concerned with photosynthesis or chlorophyll, which also connects with the zma00196 pathway. Most of the genes in the pathways zma01100, zma01110, and zma00196 (photosynthesis-antenna proteins) were down-regulated, whilst seven genes (GRMZM2G429118, GRMZM2G057281, GRMZM2G 162529, GRMZM2G436986, GRMZM2G108514, GRMZ M2G035213, and GRMZM2G103773) were up-regulated at V9. The expression of most DEGs in these pathway reached their lowest levels at stage V6 (Fig. 8). These findings demonstrate that the response to infection with RBSDV mainly involved multiple maize pathways, such as zma01100, zma01110 and zma00196.

## Discussion

### RBSDV-responsive miRNAs and target genes in maize

Many studies have reported that small RNAs play regulatory roles in the expression of numerous genes in plants during plant growth, development, and stress responses (Jones-Rhoades *et al.*, 2006; Liu *et al.*, 2014*a*; Xu *et al.*, 2014; Yan *et al.*, 2015). Some studies have been published on the role of small RNAs in plant responses to infection with viruses (Bazzini *et al.*, 2007; Tagami *et al.*, 2007; Singh *et al.*, 2012; Xiao *et al.*, 2014; Xu *et al.*, 2014), but only a few in the family *Reoviridae*. A previous study used deep sequencing to characterize the small RNA profiles of rice plants infected with *Rice dwarf virus* (RDV) or RSV (Du *et al.*, 2011). Seven putative novel miRNAs (pn-miRNAs) with previously uncharacterized precursor sequences were identified in rice in response to RSV infection (Guo *et al.*, 2012). Microarray profiling of rice miRNAs expressed in response to *Southern rice black-streaked dwarf virus* (SRBSDV) identified 56 miRNAs that were regulated in response to this disease in rice plants. Analyses of target genes indicated that the expression of four miRNA families (miR164, miR396, miR530, and miR1846) was positively or negatively correlated with that of their respective targets, genes that were associated with symptom development. However, the expression of other miRNAs was not correlated with the expression of other genes, which implies that other circumstances can affect the interactions between miRNAs and their target genes (Xu *et al.*, 2014). The miRNAs and their target genes expressed in response to RBSDV were characterized in rice leaves and roots at 1 month following inoculation with the virus (Sun *et al.*, 2014). In that study, deep sequencing revealed that the expression of 14 miRNAs in leaves and 16 miRNAs in roots was altered significantly during the response to RBSDV infection in rice (Sun *et al.*, 2014). However, the roles of miRNAs and their target genes in response to RBSDV inoculation in an early maize developmental stage (V3) have not previously been reported. In our study, combined analysis of the expression of miRNAs and their target genes by transcriptome sequencing revealed RBSDV-response pathways in maize. Three non-conserved and 28 conserved miRNAs representing 17 known miRNA families and 14 novel miRNAs were identified among small RNA sequence data (Supplementary Table S2). The expression dynamics of three miRNA families in response to RBSDV at the maize V3 stage were revealed.

In the first group of miRNAs, seven known miRNA families (miR1432, miR319, miR408, miR4366, miR8155, miR9773, and miR9774) were up-regulated in the maize library BP3–BN3. In rice infected with RBSDV at a later developmental stage, the expression of the miR1432 family increased in the leaves but decreased in the roots (Sun *et al.*, 2014). However, the expression of the miR408 family was reduced in both leaves and roots (Sun *et al.*, 2014). In addition, other miRNAs that are differentially expressed at the maize V3 stage, such as miR319, miR4366, miR8155, miR9773, and miR9774, were not detected in the later leaf and root stages in rice. These discrepancies may be species-specific responses in maize and rice, or due to differences in sampling time points after RBSDV infection. miR1432 is thought to be involved in pollen development and male sterility (Yan *et al.*, 2015) as well as in drought shock stress (Kantar *et al.*, 2011) in rice. The target gene of miR319, the transcription factor TCP1, is related to the S RNA (NSs) of the *Groundnut bud necrosis virus* (GBNV) in tomato (Goswami *et al.*, 2012). Leaf-curling symptoms in plants infected with *Tomato leaf curl virus* (ToLCV) might also be associated with miR319 (Naqvi *et al.*, 2010). Other targets of miR319 might also be involved in the thickening of cell walls in the fibrous bast material during the elongation phase in ramie (*Boehmeria nivea*; Wang *et al.*, 2014), and in cell proliferation (Schommer *et al.*, 2014), stress-response regulation (Sunkar and Zhu, 2004), and photomorphogenesis in Arabidopsis (Tsai *et al.*, 2014). Previous studies have connected miR408 with responses to plant pathogens and symbionts, such as infection with *Puccinia graminis* f. sp. *tritici* in *Triticum aestivum* (Gupta *et al.*, 2012), *Cotton leaf curl Allahabad virus* (CLCuAV) in cotton (Khan, 2014), or *Exserohilum turcicum* in maize (Wu *et al.*, 2014), and symbiosis with beneficial diazotrophic bacterial endophytes in maize (Thiebaut *et al.*, 2014). For RBSDV-infected maize, the seven up-regulated known miRNA families might affect the expression of MRDD symptoms and the development of maize cells in response to RBSDV infection.

In the second group of miRNAs, the expression of five miRNA families (miR156, miR158, miR160, miR395, and miR8677) was down-regulated in response to RBSDV infection in maize. In rice infected with RBSDV at later stages, the expression of miR156 increased in leaves and roots (Sun *et al.*, 2014). The regulation of miR156 expression is apparently related to the improvement of plant architecture in rice (Chen *et al.*, 2015), *Solanum tuberosum* ssp. *andigena* (Bhogale *et al.*, 2014), and *Lotus japonicus* (Wang *et al.*, 2015); control of flowering time in Arabidopsis and other plants (Wang, 2014); and growth of individual organs and whole plants in Arabidopsis (Chew *et al.*, 2014). A previous study demonstrated that miR158 might act in male sterility in *Brassica campestris* ssp. *chinensis* (Jiang *et al.*, 2013). miR160 also regulates gene expression and activity cascades during the early stages of plant development (Nonogaki, 2010), pathological development of stem canker disease in *Populus trichocarpa* (Zhao *et al.*, 2012), symbiotic nodule development and auxin sensitivity in soybean (Turner *et al.*, 2013), and the response to *Soybean mosaic virus* (SMV) (Yin *et al.*, 2013). The predicted targets of miR395 might be involved in morphological and metabolic adaptations in maize root cells (Zhang *et al.*, 2008) and in wheat response to infection with *Wheat streak mosaic virus* (WSMV) (Fahim *et al.*, 2012). The results of the present study showed that down-regulation of miR156, miR158, miR160, and miR395 upon RSBVD infection could be related to plant architecture in maize.

In the third group of miRNAs, members within five known miRNA families (miR166, miR167, miR169, miR396, and miR399) increased or decreased at the same time. miR166, miR167, miR169, and miR396 also responded to RBSDV in rice leaves and roots (Sun *et al.*, 2014). Previous results indicated that miR166 controls development of the shoot apical meristem in Arabidopsis (Zhu *et al.*, 2011), the nodule in *Medicago truncatula* (Boualem *et al.*, 2008), and cell wall thickening in bast during the elongation phase in ramie (Wang *et al.*, 2014). Targets of miR167 include an auxin response factor that leads to floral development defects and female sterility in tomato (Liu *et al.*, 2014*b*), plant growth retardation upon infection with *Hibiscus chlorotic ringspot virus* (HCRSV) (Gao *et al.*, 2013), and decreased response to RBSDV in rice (Sun *et al.*, 2014). The NF-YA genes, which are targets of miR169, have been closely associated with stress-induced flowering, abiotic stress response in leaves or roots (Calvino and Messing, 2013; Borowski *et al.*, 2014; Sorin *et al.*, 2014; Luan *et al.*, 2015) and with root nodule development (Reynoso *et al.*, 2012). In our study, the expression of miR169l-5p changed with a log_2_FC value of 1.3689, which is consistent with results of a previous study in rice (Sun *et al.*, 2014). Its target gene, which was annotated as nuclear factor Y, subunit A6, was negatively regulated. While the expression of miR169i-p5 decreased with a log_2_FC value of −1.2605, its major target genes, which were annotated as nucleolin (suppressor of Mek), a serine/threonine-protein kinase Rio1, a member of the 60S acidic ribosomal protein family, and ubiquitin-related enzymes, were negatively regulated. A few target genes were positively regulated, and were annotated as an abscisic acid stress- and ripening-related gene, a UDP-glycosyltransferase superfamily protein, and an ATPase. miR396 is related to the regulation of stress responses (Liu *et al.*, 2008) and pistil development in Arabidopsis (Liang *et al.*, 2014), and to arsenate and arsenite stress responses in wild accessions of rice (Sharma *et al.*, 2015). miR399 was isolated from the phloem of *Brassica napus* (Buhtz *et al.*, 2008), and is related to the expression of Huanglongbing (HLB, or citrus greening) disease symptoms in citrus (Zhao *et al.*, 2013), and is also to the function of a MYB transcription factor in signaling phosphorus deficiency (Valdes-Lopez *et al.*, 2008). Combined analyses indicated that the regulation of the miRNA families miR166, miR167, miR169, miR396, and miR399 might be involved in maize tissues and stress responses. The differential expression patterns observed within a single miRNA family need to be elucidated in future studies.

### Analysis of dwarfing symptoms associated with infection by RBSDV

**Fig. 9.**
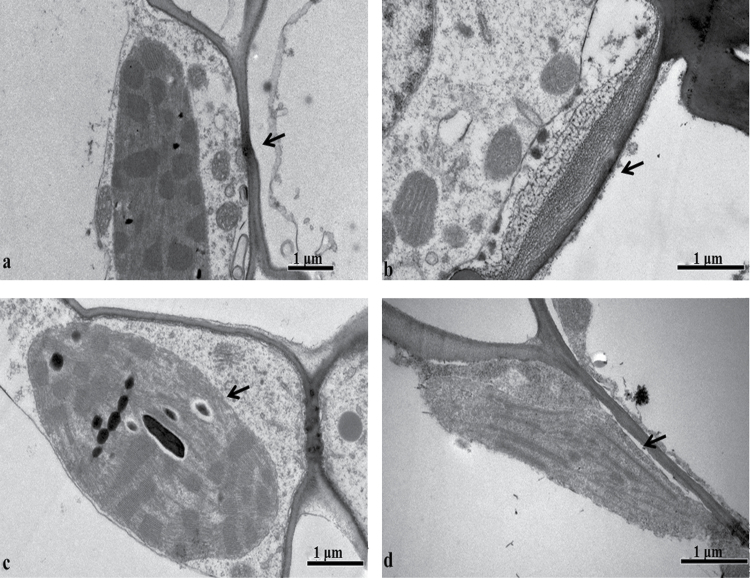
Morphological changes in cells in cross-sections of RBSDV-infected maize leaf tissue near leaf veins. (a) Cell walls in an uninfected maize leaf.(b) Cell walls in a diseased maize leaf. (c) Chloroplasts in an uninfected maize leaf. (d) Chloroplasts in a diseased maize leaf. Arrows in (a) and (b) point to the cell wall; arrows in (c) and (d) point to chloroplasts.

Plant height is an important agronomic trait that is influenced by biosynthetic and metabolic pathways related to cell walls (Reiter *et al.*, 1993; Kalluri *et al.*, 2014), and it is negatively affected by MRDD. The cytological characteristics of leaf tissues from diseased B73 plants infected with RBSDV by viruliferous SBPH and from healthy B73 plants were investigated by electron microscopy. The cell walls of diseased B73 leaves were changed by infection and appeared bulky compared with those of healthy B73 leaves (Fig. 9a, b). Previous studies have shown that members of the cellulose synthase (CESA) superfamily including CESA active subunits and CESA-like (CSL) proteins are important in cell wall biosynthesis at the stage when white streaks or enations appear on the uppermost newly expanded leaf (Jia *et al.*, 2012). Other studies have indicated that the expression of several CESA genes and some CSL genes significantly decrease in response to infection with RSV, RDV, or RBSDV, all of which belong to the *Reoviridae* (Shimizu *et al.*, 2007; Satoh *et al.*, 2010; Jia *et al.*, 2012). It has previously been showed that normal cell wall biosynthesis and plant growth are closely related to CSL D4 (Li *et al.*, 2009). In the present study, the expression of CESA3 (GRMZM2G025231) and CSLF6 (GRMZM2G122277 and GRMZM2G110145) genes were all decreased, consistent with the behavior of their previously known homologs. The plant cell wall is composed largely of cellulose, lignin, and pectin (Kalluri *et al.*, 2014). Our results showed that the expression of two lignin-related genes, the laccase precursor proteins, and most pectinesterase genes increased in response to RBSDV infection in maize. Combining the results of the present study with those of previous ones, the dwarf symptom of MRDD could be due to changes in cell wall-related structure and biosynthesis, possibly attributable to the decreased expression of CESA and CSLF genes.

### Analysis of the pathogen-response pathway as related to the ubiquitin pathway

A recent study illustrated that the ubiquitin-proteasome system of papaya is modified upon the infection of plants with the dsRNA *Papaya meleira virus* (PMeV) (Abreu *et al.*, 2015). Previous results suggested that a core subunit of E3 ubiquitin ligase, *Z. mays* SKP1 (SKP1^Maize^), strongly interacts with P7-2, a non-structural protein of RBSDV (Wang *et al.*, 2013). In the present study, the expression of ubiquitin-related genes was altered significantly among miRNA target genes and the transcriptome. Three negatively regulated genes (GRMZM2G087312, GRMZM2G094595, and GRMZM2G012690), which are targets of miR169i-p5, are ubiquitin-related genes, and include NADH: ubiquinone oxidoreductase, ubiquitin-conjugating enzyme, and the ubiquitin-associated (UBA)/TS-N domain-containing protein. Based on target prediction, three transcripts of GRMZM2G027546 that are targets for miR8155 are ubiquitin-conjugating enzyme 25. Three ubiquitin biosynthesis-related DEGs (GRMZM2G046848, GRMZM2G144782, and GRMZM2G303964) were detected in response to RBSDV infection. GRMZM2G303964 is also related to the binding GO term (GO:0005488), which includes the negatively regulated gene GRMZM069316 that is a target of miR169i-p5. GO analysis revealed that these three DEGs are related to the ubiquitin ligase complex (GO:0000151), ubiquitin-protein ligase activity (GO:0004842), and protein ubiquitination (GO:0016567). GRMZM2G144782 probably takes part in the ubiquitin-mediated proteolysis pathway (zma04120 in KEGG). The results above suggest that ubiquitin-related genes and genes involved in the ubiquitin biosynthesis pathway might be influenced during maize infection with RBSDV. Future studies will be needed to elucidate the mechanism of interaction between the maize ubiquitin pathway and RBSDV infection.

### Analysis of the pathogen-response pathway in terms of chloroplast and photosynthetic functions

Previous research has indicated that chloroplasts can be affected by infection of plants with viruses such as TMV (Chiba and Tominaga, 1952; Reinero and Beachy, 1986; Bhat *et al.*, 2013), *Cucumber mosaic virus* (CMV) (Mochizuki *et al.*, 2014), or RSV (Xu and Zhou, 2012; Kong *et al.*, 2014). Recent research suggests that the non-structural protein P5-2 can be specifically targeted to chloroplasts (Liu *et al.*, 2015). In the present study, the chloroplasts of healthy B73 plants developed normally, with normal ultrastructure and clearly defined internal matrix, grana, thylakoids, and starch grains (Fig. 8c). Internal and external chloroplast structures changed or vanished upon infection with RBSDV (Fig. 8d). Transcriptome analysis showed that the expression of several chloroplast-related genes was altered, and that the expression of photosynthesis- and photosynthetic pigment-related genes including chlorophyll and carotenoids was down-regulated in maize plants infected with RBSDV (Supplementary Table S6). The decreased expression of these photosynthesis-related genes seems to indicate that chloroplasts and photosynthesis were affected during the early stages of RBSDV infection in maize. When symptoms of RBSDV infection appear, the chloroplast- or photosynthesis-related genes could not be detected, although this could have been due to the sampling time point.

The negatively regulated gene GRMZM2G031169 referred to three GO terms (GO:0003824, GO:0044237, and GO:0050662). Fifty-one significant DEGs were detected for GO:0003824, which is annotated as catalytic activity. Among these, the expression levels of three alpha-amylase precursors (GRMZM2G070172, GRMZM2G074781, and GRMZM2G138468) were all increased relative to control plants, with log_2_FC values of 3.3815, 1.9730, and 1.3342, respectively. However, the expression levels of three chloroplast precursor genes (GRMZM2G046163, GRMZM2G015892, and GRMZM2G097457) were lower in RBSDV-infected leaves. Previous research results indicated that alpha-amylase is the only enzyme that can degrade starch granules in spinach chloroplasts (Steup *et al.*, 1983). In rice leaves, alpha-amylase isoform I-1 is involved in starch degradation through the endoplasmic reticulum–Golgi system (Asatsuma *et al.*, 2005). During biotic and abiotic stress, an alpha-amylase (At4g25 000) was induced and secreted in Arabidopsis leaves (Doyle *et al.*, 2007). In *Plasmopara viticola*-infected grapevine leaves, increased alpha-amylase activity was involved in the starch degradation pathway (Gamm *et al.*, 2011). In the present study, starch grains were not apparent in the chloroplasts of diseased maize plants compared with those of healthy control plants (Fig. 8c, d). The disappearance of starch grains may be related to the altered expression of alpha-amylase precursor genes. The expression of several miRNAs and other genes detected in the transcriptome related to catalytic activity, cellular metabolic processes, and coenzyme binding responded to RBSDV infection in maize. These genes encoded enzymes including 3-ketoacyl-CoA synthase, alpha-amylase precursor, AMP-binding enzyme, dehydrogenase, phosphatase, reticuline oxidase-like protein, UDP-glucuronate 4-epimerase, and others. We propose here that the degradation of starch grains in the chloroplast, decreased expression of photosynthesis-related genes, and the deformation of chloroplasts are closely related to infection of maize plants by RBSDV.

Seventeen DEGs related to three KEGG pathways (zma01100, zma01110, and zma00196) showed differential expression from growth stages V3 to V12. These DEGs mainly take part in zma01100 and zma01110, which means metabolic pathways and biosynthesis of secondary metabolites. Metaboism of terpenoids and polyketides, metabolism of cofactors and vitamins, energy metabolism, carbohydrate metabolism, and nucleotide metaboism were directly involved. Some DEGs in zma01100 and zma01110 also participate in carbon fixation in photosynthetic organs and in chlorophyll A–B binding proteins. These results were consistent with analysis of miRNA-target genes and the transcriptome. Pathways that related to photosynthetic organs and chlorophyll A–B binding proteins showed significant differential expression at five growth stages. The majority of DEGs showed their lowest expression at stage V6 with higher levels at V9. These results may suggest that V6 is the sensitive growth stage of maize in relation to its response to infection by RBSDV.

## Supplementary data

Supplementary data are available at *JXB* online.

Figure S1. Quantitative real-time RT-PCR (qRT-PCR), plant disease status, and cytological identification of the infection status of maize inoculated with RBSDV.

Table S1. Primers used for virus detection by qRT-PCR.

Table S2. Differentially expressed miRNAs and target genes.

Table S3. Three non-conserved and 28 conserved miRNAs from virus-infected or virus-free leaves at growth stage V3.

Table S4. Fourteen novel miRNAs from virus-infected or virus-free leaves at growth stage V3.

Table S5. Differentially expressed target genes for known miRNAs.

Table S6. Differentially expressed target genes for novel miRNAs.

Table S7. Alterations in expression of genes at two time points in maize infected with RBSDV.

Table S8. Alterations in expression of cell-related genes in maize infected with RBSDV.

Table S9. Alterations in expression of chloroplast-related genes in maize infected with RBSDV.

Table S10. Alterations in expression of chloroplast-related genes in maize infected with RBSDV.

Table S11. GO terms for GRMZM2G069316.

Table S12. GO terms for GRMZM2G031169.

Table S13. qRT-PCR primers used in this study.

Supplementary Data

## Abbreviations:

MRDD: maize rough dwarf disease
RBSDV: *Rice black-streaked dwarf virus*
SBPH: small brown planthopper
RSV: *Rice stripe virus*
qRT-PCR: quantitative real-time PCR
DAI: days after inoculation
DEGs: differentially expressed genes
CSL: CESA-like.
