# Supplementary material for: Dual transcriptome analysis reveals insights into the response to Rice black-streaked dwarf virus in maize
Source: J Exp Bot. 2016 Jul 18;67(15):4593–609. doi: 10.1093/jxb/erw244 (PMC4973738; doi:10.1093/jxb/erw244)
Supplement: Supplementary Data [file supp_67_15_4593__index.html]

Dual transcriptome analysis reveals insights into the response to Rice black-streaked dwarf virus in maize — Dual transcriptome analysis reveals insights into the response to Rice black-streaked dwarf virus in maize — Supplementary Data 

# Dual transcriptome analysis reveals insights into the response to *Rice black-streaked dwarf virus* in maize

## Supplementary Data

Data files

- Supplementary\_Figure\_1.pdf - Supplementary Data
- Supplementary\_Table\_S1\_S6.xlsx - Supplementary Data
- supplementary\_tables\_S7\_S13.xlsx - Supplementary Data
