# Supplementary material for: Dual transcriptome analysis reveals insights into the response to Rice black-streaked dwarf virus in maize
Source: J Exp Bot. 2016 Jul 18;67(15):4593–609. doi: 10.1093/jxb/erw244 (PMC4973738; doi:10.1093/jxb/erw244)
Supplement: Supplementary Data [file supp_erw244_Supplementary_Figure_1.pdf]

Dual transcriptome analysis reveals insights into the response to Rice black-streaked dwarf virus in maize.

Yu Zhou, Zhennan Xu, Canxing Duan, Yanping Chen, Qingchang Meng, Jirong Wu, Zhuanfang Hao, Zhenhua Wang, Mingshun Li, Hongjun Yong, Degui Zhang, Shihuang Zhang, Jianfeng Weng, and Xinhai Li

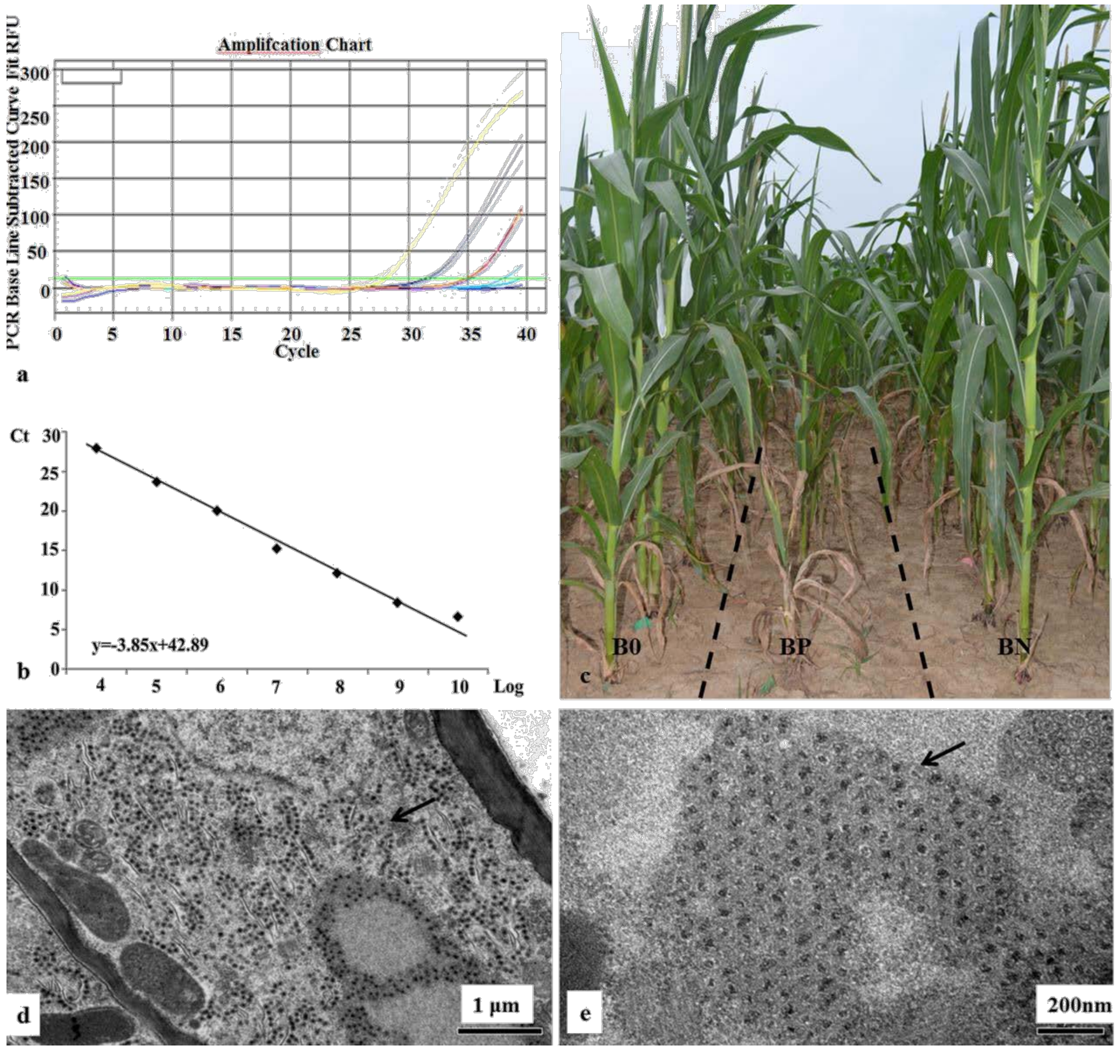

Supplementary Fig. 1S. Quantitative real-time RT-PCR (qRT-PCR), plant disease status, and cytological identification of the infection status of maize inoculated with RBSDV.

a. Amplification plot of RBSDV by qRT-PCR, the curved lines from left to right represent three technology replications of 20.0 d, 6.0 d, 3.0 d, 1.5 d, 0 d.

b. The standard curve for real-time RT-PCR.

c. B73 with MRDD for control, viruliferous SBPH, and virus-free SBPH treatments.

d. Virus in the cytoplasm of leaf.

e. Crystallized viral particles in leaf cell. B0, control; BP, B73 inoculated with viruliferous SBPH; BN, B73 inoculated with virus-free SBPH. Arrows point to RBSDV particle.
